# Capsaicin exerts synergistic pro-apoptotic effects with cisplatin in TSCC through the calpain pathway via TRPV1

**DOI:** 10.7150/jca.98075

**Published:** 2024-07-09

**Authors:** Xin-Yue Zhou, Qi-Wei Zhao, Zhuang Li, Xia-Yang Liu, Yu Wang, Feng-Hua Wu, Min Zhao, Yan-Mei Zhang, Gang Zhao, Guo-Hua Yang, Xiao-Hong Guo

**Affiliations:** 1 Department of Basic Medicine, Hubei University of Chinese Medicine, Wuhan 430065, China.; 2 Hubei Shizhen Laboratory, Wuhan, Hubei 430065, China.; 3 Department of Medical Genetics, School of Basic Medical Science, Demonstration Center for Experimental Basic Medicine Education, Wuhan University, Wuhan 430071, China.

**Keywords:** capsaicin, cisplatin, TRPV1, calpain pathway, TSCC

## Abstract

Capsaicin (CAP) exerts significant anti-tumor effects on a variety of tumors, with low intrinsic toxicity. Cisplatin (DDP) is currently the first-line drug for the treatment of oral cancer; however, its clinical efficacy is impeded by chemoresistance and negligible side effects. Whether the combined use of CAP and DDP has a synergistic antitumor effect on tongue squamous cell carcinoma (TSCC) cells and its underlying mechanisms remains unclear. The present study revealed that CAP reduced the activity of TSCC cells in a dose- and time-dependent manner. We also observed changes in the mitochondrial functional structure of TSCC cells, along with the induction of mitochondrial apoptosis. Moreover, when CAP was combined with DDP, a synergistic cytotoxic effect on TSCC cells was observed, which had a significant impact on inducing apoptosis, inhibiting proliferation, and disrupting the mitochondrial membrane potential in TSCC cells compared to the single-drug treatment and control groups. These effects are associated with TRPV1, a high-affinity CAP receptor. The combined use of CAP and DDP can activate the TRPV1 receptor, resulting in intracellular Ca^2+^ overload and activation of the calpain pathway, ultimately leading to mitochondrial apoptosis. This potential mechanism was validated in TSCC xenograft models. In conclusion, our findings clearly demonstrate that CAP exerts synergistic pro-apoptotic effects with DDP in TSCC through the calpain pathway mediated by TRPV1. Thus, CAP can be considered an effective adjuvant drug for DDP in the treatment of TSCC.

## Introduction

Oral cancer (OCC) has a high incidence and mortality rate and is ranked as the sixteenth most prevalent malignant tumor worldwide 1. In 2020, lip and oral cancers caused 177,757 deaths and 377,713 incident cases worldwide in 2020 2. Owing to the widespread habits of chewing betel nuts, cigarette smoking, and alcohol consumption, oral cancer is particularly common in South and Southeast Asia. Oral cancer mainly occurs on tongue (ventral margin, 40 % of cases), the floor of the mouth (30 % of cases), and the lower lips 3. Compared to other tumor sites, tongue squamous cell carcinoma (TSCC) is the most malignant and migratory of oral cancer 4. Surgery is the primary treatment for TSCC, followed by radiotherapy, chemotherapy, targeted drug therapy and immunotherapy 5. However, it still has the potential for recurrence or metastasis, and the 5-year survival rate of patients is approximately 50 % 6.

Cisplatin (cis-diamminedichloroplatinum II, DDP) is a well-known first-line chemotherapeutic drug for numerous human solid tumors, including small cell lung cancer, gastric cancer, bladder cancer, ovarian cancer, and TSCC 3, 7. In brief, the main mechanism of action of DDP involves intra-strand DNA cross-linking between adjacent purines, interfering with DNA repair mechanisms, causing DNA damage, subsequently inhibiting tumor cell invasion, and inducing apoptosis 7. Besides, it can also target mitochondrial DNA (mtDNA) to induce apoptosis 7, 8. However, chemoresistance and negligible side effects impede the clinical efficacy of DDP and seriously affect health and quality of life 9. Therefore, it is imperative to develop an effective and safe treatment strategy that combines low-dose DDP with other drugs to reduce the DDP-related side effects and chemoresistance.

Phytochemicals are a range of chemicals that are extracted and purified from plants, including fruits, vegetables, spices and grains 10. Recent research has shown that the anti-tumor properties of phytochemicals occur primarily in cancer cells, with low intrinsic toxicity in normal cells, which has attracted significant interest 11. Capsaicin (trans-8-methyl-N-vanillyl-6-nonenamide, CAP), a derivative of homovanillic acid, is the main pungent ingredient in chili pepper 12. CAP and its analogs have been used medicinally for centuries owing to their analgesic, antioxidant, anti-inflammatory, and anti-obesity properties. More recently, they have garnered considerable attention for their notable anti-tumor effects in different types of malignancies, such as renal, gastric, and breast cancer 13-15, as well as their ability to enhance the effects of conventional chemotherapeutic agents through synergistic mechanisms 16. Capsaicin combined with cisplatin has been found to have a synergistic anti-tumor effect on human osteosarcoma 11.

The ability of CAP to produce "heat sensation" is mediated by the transient receptor potential vanilloid 1 (TRPV1) receptor, which belongs to the transient receptor potential (TRP) superfamily of cation channel receptors of high affinity agonists. The TRPV receptor family consists of six members (TRPV1-6), and CAP is a high-affinity TRPV1 receptor agonist 17. Studies have shown that TRPV1 can be activated by CAP, temperature, and low pH. Activation of TRPV1 leads to an influx of Na^+^ and Ca^2+^ from outside the cell, and excessive amounts of intracellular Na^+^ and Ca^2+^ lead to cell death 18-20. The apoptotic activity of CAP has been found to be mediated by TRPV1 receptors in colorectal cancer cells, uroepithelial cancer cells, and human osteosarcoma cells 21-24. In addition, DDP can overcome drug resistance by blocking the autophagy-mediated EGFR signaling pathway overactivation through TRPV1 receptors 25. Therefore, based on the above research, we speculated that CAP and DDP in TSCC may have a synergistic anti-tumor effect and may be related to the TRPV1 receptor.

However, the mechanisms underlying the apoptotic activity of CAP in TSCC have not been fully elucidated, and whether CAP enhances the anti-tumor effect of DDP in TSCC requires further study. Therefore, this study aimed to investigate the effects of CAP alone or in combination with DDP on TSCC both *in vitro* and *in vivo,* and to explore the underlying molecular mechanisms.

## Methods

### Cell culture and reagents

SCC9 (RRID: CVCL_1685), Cal27 (RRID: CVCL_1107) and HN6 (WSU-HN6, RRID: CVCL_5516) cell lines are all human Tongue squamous cell carcinoma. HOK (HOK-16A, RRID: CVCL_B404) is human normal oral keratinocyte cell line, SCC9 and Cal27 cells were purchased from American Type Culture Collection (ATCC, Manassas, VA, USA). HOK and HN6 cells were generously provided by the Stomatological Hospital Affiliated to Shanxi Medical University in September 2022. Cal27 and HN6 were cultured in Dulbecco's modified Eagle's medium, DMEM High Glucose (Biosharp, Beijing, China), supplemented with 10 % fetal bovine serum(FBS) (Excell, Shanghai, China), and 1 % Penicillin-Streptomycin Solution (Biosharp, Beijing, China). SCC9 cells were maintained in Dulbecco's Modified Eagle Medium/Nutrient Mixture F-12, DMEM/F12 (Biosharp, Beijing, China), containing 1 % L-Glutamine, 0.5 mM Sodium Pyruvate, HEPES (Biosharp, Bejing, China), 0.4 μg/mL hydrocortisone (BBI Life Science, Shanghai, China), 10 % FBS and 1 % Penicillin-Streptomycin Solution (Biosharp, Beijing, China). HOK cells were grown in RPMI-1640 medium (Biosharp, Beijing, China) containing HEPES, 10 % FBS and 1 % Penicillin-Streptomycin Solution. All of these cells were incubated at 37 °C in humidified surroundings of 5% CO_2_.

Capsaicin (CAP), a TRPV1 receptor agonist, Ruthenium red (RR), a TRPV generalized antagonist 26, BAPTA-AM, a well-known membrane permeable Ca^2+^ chelator and Capsazepine (CPZ), an antagonist of TRPV1 receptor, all of them were obtained form MedChem Express, Monmouth Junction, NJ, USA and prepared at 100 mM and 10 mM stock solution in DMSO, respectively. The total final DMSO concentration in the medium was less than 0.1 %. (volume per volume). DDP (MedChem Express, Monmouth Junction, NJ, USA) was dissolved in normal saline at 1 mg/ml. Prior to the experiment, the stock solutions were filter-sterilized by a 0.2 mM pore filter. All the stock solutions were stored at - 20 °C after aliquoting.

Rabbit anti-TRPV1 polyclonal antibody (DF10320, 1:1000) was purchased from Affinity Biosciences (Melbourne, Australia); Rabbit anti Bcl-2 (P65) polyclonal antibody (BS1511, 1:1000) , rabbit anti Bax polyclonal antibody (BS61098, 1:1000), rabbit anti Cytochrome c (Cyt c) polyclonal antibody (BS61098, 1:1000), rabbit anti Caspase-3 polyclonal antibody (BS6428, 1:1000), rabbit anti Caspase-7 polyclonal antibody (BS6544, 1:1000), rabbit anti activated-Caspase-9 polyclonal antibody (BS7070, 1:1000), rabbit anti GAPDH (AP0063, 1:18000) and goat anti-rabbit IgG (H + L)-HRP secondary antibodies (BS13278, 1:20000) were purchased from Bioworld Technology (Minnesota, USA).

### Cell viability assay

Cell viability was measured by Cell Counting Kit-8 (CCK-8) assays. HOK, Cal27, SCC9 and HN6 cells (5 × 10^3^ cells/well) were seeded in 96-well plates and incubated for 24 h to adhere. Then cells were exposed to different doses of CAP and/or DDP for 24 h, 36 h or 48 h. After treatment, a 10 μL aliquot CCK-8 solution was dissolved in culture medium, then supplemented into each well for an additional hour. After that, the plates were put in a microplate reader to measure the absorbance at 450 nm. This formula used to determine cell viability: cell viability (%) = (experimental group absorbance value - blank group absorbance value) / (control group absorbance value - blank group absorbance) × 100 %. The synergistic effects of DDP and CAP on TSCC cells were analyzed by the CompuSyn program (Biosoft, USA). The combined effect is divided into three categories: synergistic effect (combination index, CI < 1), additive effect (CI = 1), and antagonistic effect (CI > 1).

### Colony formation assay

Cal27 and HN6 cells were seeded into 6-well plates at a density of 1000 cells/ well for 3 days at 37 °C. After that, replaced the medium containing different doses of CAP and/or DDP for 6 h, then changed the new complete medium incubated for two weeks, replaced the medium every 3 days during this time. In accordance with a prior demonstration, colonies were fixed with 4 % paraformaldehyde for 15 min at room temperature and then stained with 0.1 % crystal violet for 20 min. ImageJ 2.3.0. (National Institutes of Health), was used to count the number of clones.

### Cell apoptosis assay

Apoptotic effects were determined by flow cytometry using an Annexin V-FITC/PI Apoptosis Kit (Bestbio, China). Cells (1 × 10^6^ cells/well) were seeded in 6-well plates for 24 h and allowed to adhere. Then exposed to various concentrations of CAP (100 μM, 150 μM and 200 μM) and/or DDP (4 μg/mL) for 24 h once the density reached 70 %. After treatment, the cells were collected using trypsin without EDTA (Biosharp, China), centrifuged at 2000 rpm for 5 min at room temperature, and washed thrice with ice-cold phosphate-buffered saline (PBS) to create single-cell suspensions. Finally, we resuspended the single cell suspensions with binding buffer (400 μL) and stained with 5 μL Annexin V-FITC and 10 μL PI for 30 min away from light. All samples were analyzed using a flow cytometer (BD Biosciences, Franklin Lakes, NJ, USA).

### Dual Acridine orange (AO)/ethidium bromide (EB) fluorescent staining assay

AO/EB double fluorescence staining was used to observe morphological changes in cells and to demonstrate the apoptotic effect. Glass slides were placed into six-well plates, then seeded with Cal27 and HN6 cells (1× 10^5^ cells/well) and allowed to adhere overnight. Next, the cells were exposed to CAP and/or DDP for 24 h. Following treatment, AO/EB double fluorescence staining solution was added to each slide and covered with a coverslip. Finally, fluorescence microscopy (model 80 i; Nikon Corporation) was used to examine morphological changes in apoptotic cells in five randomly chosen fields.

### Transmission electron microscopy (TEM)

Transmission electron microscopy was used to observe changes in the functional morphology of the mitochondrial structure after various treatments. Cal27 and HN6 cells were initially seeded in T-25 culture flask overnight at a density of 5 × 10^6^ cells for adherence. Subsequently, they were treated with CAP (200 μM) for 24 h. Following this treatment, the cells were collected and fixed with 2.5 % glutaraldehyde for 6 h at 4 °C. Subsequently, a series of graded ethanol and acetone solutions was used for dehydration. The sections were then stained with a solution containing 3 % uranyl acetate and 0.4 % lead citrate. Finally, the cellular specimens were analyzed using transmission electron microscopy (TEM) (JEM-1400 Plus, JEOL, Japan).

### Mitochondrial membrane potential measurement

JC-1 assay kit (Beyotime Biotechnology, China) was used to detect the mitochondrial membrane potential (Δψm). At a density of 1 × 10^5^ cells per well, cells were seeded in 6-well plates and incubated overnight at a density of 1 × 10^5^ cells/well. After adherence, the cells were exposed to CAP (150 μM) and/or DDP (4 μg/mL) for 24 h. Next, the cells were washed thrice with ice-cold PBS and stained with 2 mM JC-1 at 37 °C, away from light, for 20 min. The cells were washed twice with ice-cold JC-1 buffer and directly observed under a laser-scanning confocal microscope (Zeiss LSM710 META, Carl Zeiss). Five fields of view were selected randomly from each group.

### TCGA database analysis

Oral squamous cell carcinoma (OSCC) represents a prevalent malignancy within the head and neck region 27. The expression level of TRPV1 in human head and neck squamous cell carcinoma (HNSC) established in TCGA was collected from UALCAN (http://ualcan.path.uab.edu/)^28^-^29^

### Western blotting

After exposure to the aforementioned concentrations of CAP and/or DDP for 24 h, cells were washed three times with ice-cold PBS and then lysed with radioimmunoprecipitation assay (RIPA) buffer (Solarbio, China) containing protease inhibitors (Servicebio, China) on ice for 30 min to extract the total intracellular proteins. After a 15-min centrifugation at 4 °C, 12000 rpm/min, they were carefully collected and transferred the supernatant to a new tube placed on ice. The proteins were quantified using a BCA kit (YEASEN, China) prior to denaturation. Proteins (~20 μg per lane) were separated by SDS-PAGE on 7.5-15 % gels, followed by transfer onto polyvinylidene fluoride (PVDF) membranes. The membranes were then blocked with 5 % non-fat dry milk at room temperature for 90 min. Lastly, we incubated the membranes with corresponding primary antibodies at 4 °C overnight. The membranes were washed (6 min per wash) with ice-cold Tris-buffered saline containing Tween-20 (TBST) and then incubated with horseradish peroxidase-conjugated secondary antibody for 1 h at room temperature. Before immunostaining, the membranes washed thrice (7 min per wash), and protein bands were visualized by enhanced chemiluminescence (ECL) and recorded using a Bio-Rad ChemiDoc XRS.

### Lentivirus transduction

A lentiviral transfection system was used to generate TRPV1-silenced cell lines. For lentiviral construction, a mixture containing 2 µg of DNA was prepared with the ratio of lentiviral expression vector: psPAX2: pMD2G = 4:3:2 (the three kinds of plasmid were all obtained from Wuhan BioEagle Biological Science & Technology Co., Ltd). This mixture was then combined with 2 μL liposome (Neofect, China) and added to 6-well plates of HEK293T cells at a confluence of 60 %. After 6 to 12 h of transfection, the medium was replaced with fresh complete medium and the viral supernatant was harvested 48 to 72 h after transfection. The viral supernatant was filtered using a 0.45 µm pore-size filter and then infected with HN6 cells by 10 μg/mL polybrene (Solarbio, China). Following 48 h of infection, cells were selected using 0.5 µg/ml puromycin (Solarbio, China) for 2-3 weeks. The silencing efficiency was subsequently evaluated by western blotting. The lentiviral expression vector comprised a vehicle vector (shCon) and three plasmids targeting TRPV1 shRNA (obtained from Wuhan Yeakang Gene Science & Technology Co., Ltd.). The sequence of shRNAs was as follows: sh1:5'-GCTGCTGGCCTATGTAATTCT-3,' sh2:5'-GCATCTTCTACTTCAACTTCC-3,' sh3:5'-GCCTGGAGCAGCTGTTCAAGTT-3.'

### Human calpain enzyme-linked immunosorbent assay (ELISA) kit

A human calpain ELISA kit (Jingmei Biotechnology, China) was used to detect calpain activity after the different TSCC treatments. Cells were seeded onto 6-well plates at a density of 1×10^5^ per well and incubated for 70 % adherence. The cells were pre-treated with BAPTA-AM (10 μM) for 2 h, followed by the addition of CAP (150 μM) and/or DDP (4 μg/mL) co-culture for 24 h. After treatment, the cells were washed thrice with ice-cold phosphate-buffered saline (PBS) and lysed with RIPA buffer for 30 min on ice. The lysates were prepared according to the manufacturer's instructions. To measure the amount of calpain by Human calpain in each sample, the samples were placed in a microplate reader and the absorbance was measured at 450 nm. The absorbance of the untreated cells was normalized to that of the control.

### Xenograft tumor model

Twenty-five male BALB/c nude mice (4-week-old) were purchased from (Wuhan, China) and raised in the Experimental Animal Center of Hubei University of Traditional Chinese Medicine. The mice were maintained under specific pathogen-free conditions and provided with sterile feed and water daily. After adaptive rearing for 1 week, the mice were used for study initiation. They were subcutaneously injected into the right axilla, with a sterile PBS solution (200 μL) containing HN6 cells or shTRPV1 stably knockdown cell lines at a density of 1 × 10^7^ cells/mL. Treatment was initiated when the tumor volume reached 100 mm^3^. The nude mice were divided into 5 groups (5 mice in each group) as follows: 1) Blank control group, 200 μL PBS by gavage; 2) CAP group, administered 10 mg/kg CAP dissolved in 200 μL PBS by gavage; 3) DDP group, administered 4 mg/kg DDP dissolved in 200 μL PBS by gavage; 4) CAP + DDP group, administered 10 mg/kg CAP and 4 mg/kg DDP dissolved in 200 μL PBS by gavage; 5) shTRPV1 + CAP + DDP group, administered 10 mg/kg CAP and 4 mg/kg DDP dissolved in 200 μL PBS by gavage; Each group received their respective treatment every 3 days, and the tumor volume and body weight were measured for a total of 21 days. After the last treatment, the mice were sacrificed, and the necessary samples were collected. The livers and kidneys were fixed, embedded, and sectioned before staining with hematoxylin and eosin (HE). Subsequently, sections were observed and photographed under a microscope at 200 × magnification. All animal studies were approved by the Ethics Committee of Hubei University of Traditional Chinese Medicine and followed the National Institutes of Health guidelines on the care and use of animals in research 30.

### Immunohistochemistry (IHC)

Immunohistochemistry (IHC) was performed to evaluate the expression of Ki67 in xenograft tumor tissues after different treatments. Briefly, the tumor tissue was collected, fixed with 4 % paraformaldehyde, embedded in paraffin, and sliced ​​through a microtome. After the sections were dewaxed and hydrated, they were blocked with 3 % BSA (Servicebio, China) for 30 min and stained with Ki67 rabbit polyclonal antibody 1:500 (Servicebio, China) and immunohistochemistry-specific goat anti-rabbit secondary antibody 1:200 (Servicebio, China) for immunostaining. Immunoreactions were visualized with 3-3′diaminobenzidine tetrahydrochloride (brown), nuclei were counter stained by Mayer's Hematoxylin (blue). Images were captured at a magnification of 100× using a phase-contrast microscope (Nikon E100, Japan).

### TUNEL assay

According to the manufacturer's instructions, the TUNEL assay was conducted on tumor paraffin sections using TUNEL enzyme (Servicebio, China) and TUNEL label (Servicebio, China) kits to detect apoptotic effects. After incubation and sealing, slides were photographed using an upright fluorescence microscope (Nikon Eclipse C1, Japan). Five fields of view were selected randomly from each slide.

### Statistical analysis

All experiments were repeated at least thrice (n = 3) and all data were tested for normality and homogeneity of variance prior to analysis. Data are presented as Mean ± standard deviation (SD). Differences between two groups were analyzed using Student's t-test, whereas differences between single factors and multiple groups were analyzed using one-way analysis of variance (ANOVA) followed by Dunnett's post hoc test. Differences between multiple factors and groups were analyzed using two-way analysis of variance (ANOVA), followed by Tukey's post hoc test. Statistical significance was defined as * *P* < 0.05, ** *P* < 0.01, and *** *P* < 0.001. All analyses were performed using GraphPad Prism 8 software (La Jolla, CA, USA).

## Results

### Capsaicin inhibits the viability of TSCC cells but has little effect on normal oral epithelial cells

**Figure 1:**
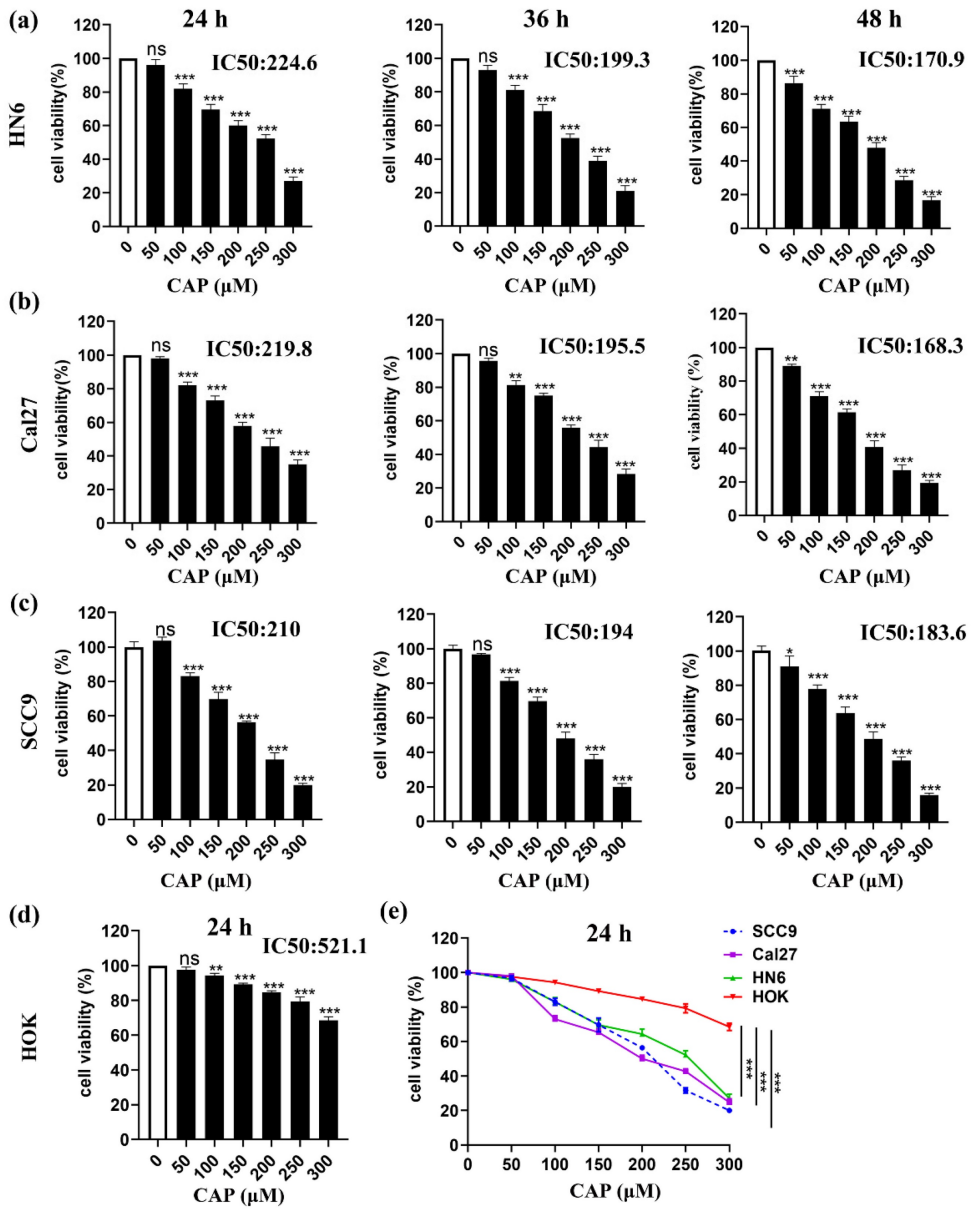
** Capsaicin (CAP) inhibited the viability of Tongue squamous cell carcinoma (TSCC) cells but has little effect on normal oral epithelial cells.** Three TSCC cells HN6 **(a)**, Cal27 **(b)** and SCC9 **(c)** were treated with varying concentrations of CAP (50, 100, 150, 200, 250 and 300 μM) for different time periods (24 h, 36 h, and 48 h), the cell viability was measured by CCK-8 assay. The normal oral epithelial cells (HOK) were exposed to different concentrations of CAP for 24 h and used CCK-8 assay to measure the cell viability **(d)**. Three TSCC cells (HN6, Cal27, SCC9) along with HOK cells were treated with CAP for 24 h, and their cell viability was assessed using the CCK-8 assay **(e)**. Data are presented as the Mean ± SD from three independent experiments. ns, not significant; **, *P* < 0.01; and ***, *P* < 0.001.

To determine the cytotoxic effect of CAP on the viability of three TSCC cell lines (SCC9, Cal27, and HN6) and normal oral cells (HOK) *in vitro*, cells were treated with varying concentrations of CAP for 24-48 h. Dose- and time-dependent inhibition of cell viability in TSCC cells was observed, as measured by the CCK-8 assay. The IC 50 values for HN6, Cal27 and SCC9 cells after 24, 36, and 48 h of CAP treatment were 224.6 μM, 199.3 μM, and 170.9 μM for HN6, 219.8 μM, 195.5 μM, and 168.3 μM for Cal27, and 210 μM, 194.0 μM, and 183.6 μM for SCC9, respectively (Fig. 1a-c). Importantly, the cell viability of HOK was still 70 % even after the maximum concentrations of CAP treatment for 24 h and the calculated IC 50 value was 521.1 μM (Fig. 1d). This suggested that the cytotoxicity of CAP in tumor cells was significantly greater than that in normal cells (Fig. 1e). Overall, our findings confirmed that CAP effectively inhibited the proliferation of TSCC cells but had only slight effect on normal oral epithelial cells.

### Capsaicin can induce apoptosis of TSCC cells through the mitochondrial pathway

**Figure 2:**
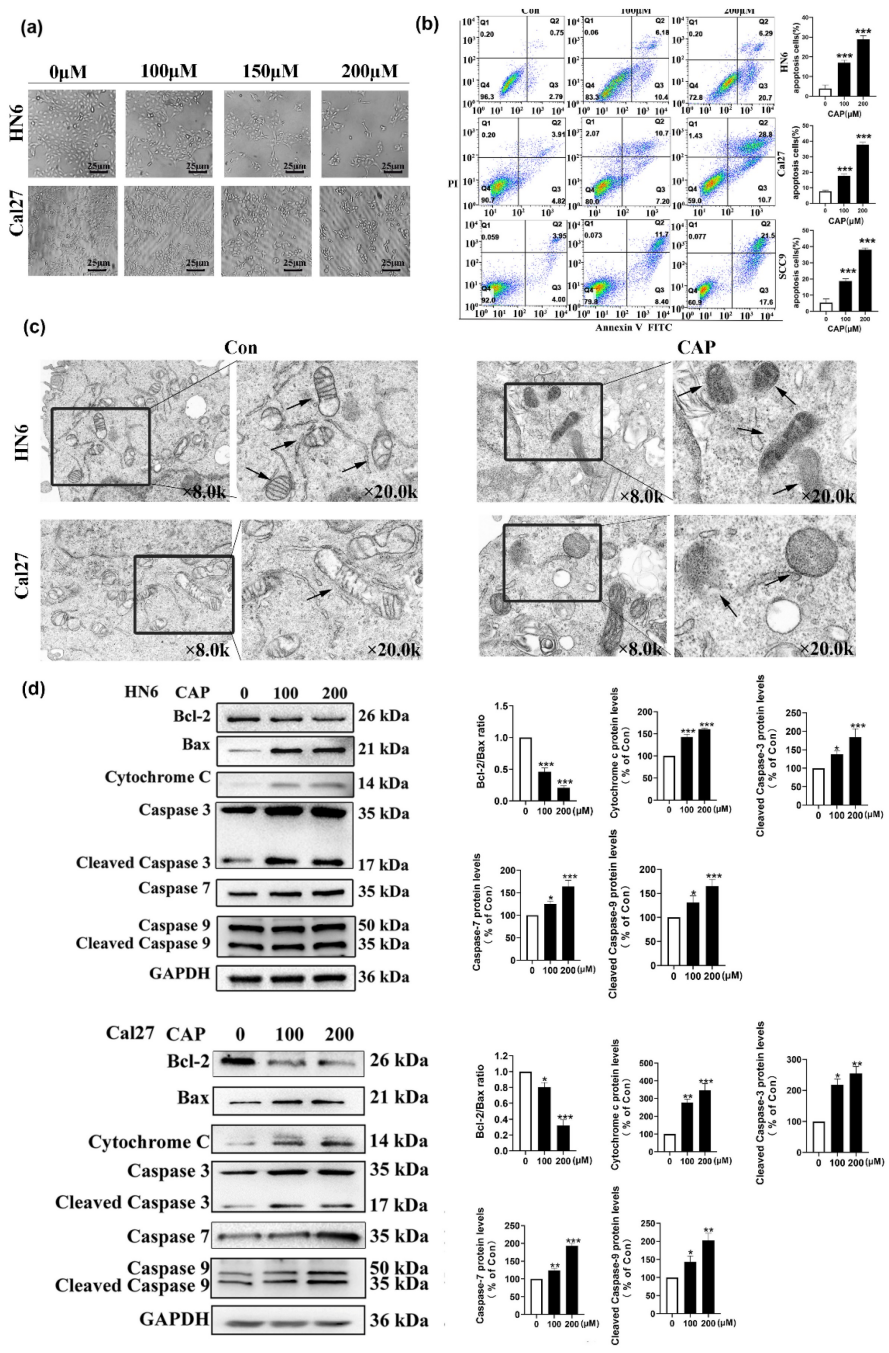
** Capsaicin (CAP) inhibited the proliferation and induced mitochondrial pathway apoptosis of Tongue squamous cell carcinoma (TSCC) cells. (a)** The changes in cell morphology of HN6 and Cal27 cells after treatment with different concentrations of CAP (100, 150 and 200 μM) for 24 h. **(b)** The apoptotic effects of three TSCC cells (HN6, Cal27, SCC9) after CAP (100 and 200 μM) treatment for 24 h were detected by Annexin V-FITC/PI double staining. The percentage of apoptotic cells were detected by the number of Q2+Q3. **(c)** Transmission electron microscopy (TEM) was employed to investigate the alterations in mitochondrial ultrastructure in Cal27 and HN6 cells following a 24 h treatment of 200 μM CAP. The arrows indicated the location of the mitochondria. The images were magnified at a scale of ×8.0k, with local magnification at ×20.0k. **(d, e)** The expression of mitochondrial apoptosis pathway related proteins in HN6 cells **(d)** and Cal27 cells **(e)** were measured by Western blotting after CAP (100 and 200 μM) treatment for 24 h. For normalization, Glyceraldehyde 3-phosphate dehydrogenase (GAPDH) was utilized. Data are presented as the Mean ± SD from three independent experiments. *, *P* < 0.05; **, *P* < 0.01; and ***, *P* < 0.001.

To investigate whether the decrease in cell viability caused by CAP was associated with the apoptotic pathway, we observed changes in cell morphology after treatment with different concentrations of CAP. The results in Fig.2a show noticeable apoptotic signs, such as cellular crinkling and reduced intercellular attachment, in HN6 and Cal27 cells. In addition, the Annexin V-FITC/PI double staining assay confirmed the apoptotic effect induced by CAP. As shown in Fig.2b, the number of apoptotic cells was significantly elevated with increasing concentrations of CAP, which indicated that apoptosis was induced by CAP in a dose-dependent manner. To determine whether the underlying mechanisms of the pro-apoptotic effects of CAP were associated with the mitochondrial pathway, TEM was used to observe the changes in the ultrastructure of the mitochondria in Cal27 and HN6 cells. After 200 μM CAP treatment for 24 h, compared with the control group, mitochondrial morphology significantly changed after CAP treatment, such as membrane rupture, cristae reduction and increased membrane density (as shown by arrows in Fig. 2c). In parallel, the expression of mitochondrial apoptosis-related proteins after CAP (100 and 200 μM) treatment were analyzed using western blotting. The results showed that the expression of the anti-apoptotic protein Bcl-2 was significantly reduced, whereas the expression of the pro-apoptotic protein Bax and the mitochondrial apoptosis-related protein Cyt c was significantly increased with increasing concentrations of CAP. Furthermore, the activity of the apoptosis-related kinases caspase-3/7/9 was upregulated. These observations suggest that CAP induces apoptosis in TSCC cells in a dose-dependent manner through the mitochondrial pathway (Fig. 2d-e).

### Capsaicin combined with cisplatin has synergistic anti-tumor effect in TSCC

**Figure 3:**
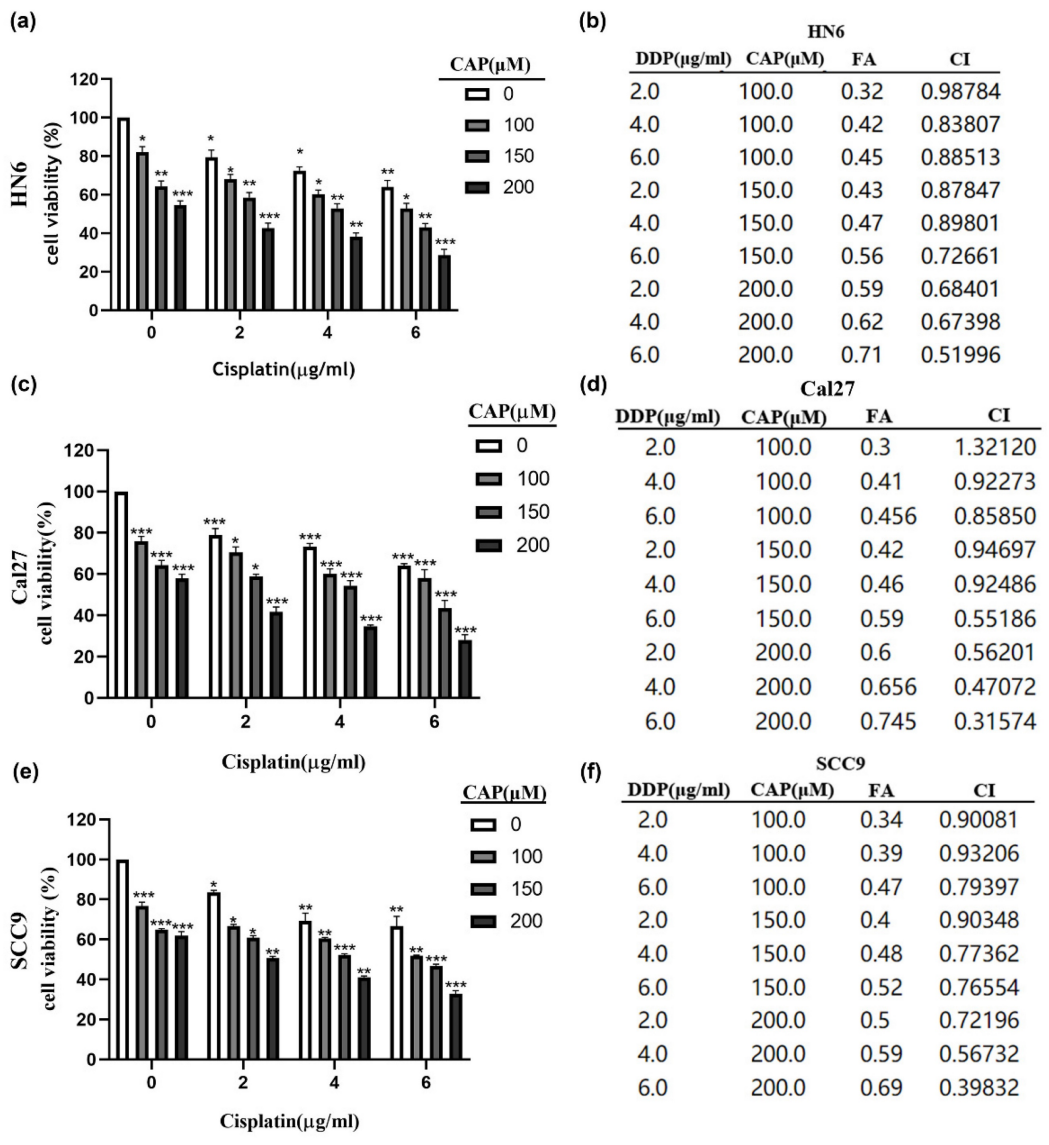
** Capsaicin (CAP) combined with Cisplatin (DDP) has synergistic cytotoxic effects in Tongue squamous cell carcinoma (TSCC) cells. (a, c, e)** Three TSCC cells HN6 **(a)**, Cal27 **(c)** and SCC9 **(e)** were treated with different concentrations of CAP (100, 150 and 200 μM) combined with DDP (2, 4, 6 μg/mL) for 24 h and the cell viability was measured by CCK-8 assay. **(b, d, f)** The combination index (CI) of CAP combined with DDP was calculated for the three TSCC cell lines, HN6 **(b)**, Cal27 **(d)**, SCC9 **(f).** Data are presented as the Mean ± SD from three independent experiments. *, *P* < 0.05; **, *P* < 0.01; and ***, *P* < 0.001.

The CCK-8 assay was performed to detect cell viability after CAP combined with DDP treatment for 24 h in three TSCC cell lines (HN6, Cal27, and SCC9). To reduce drug toxicity, we selected medium concentrations of CAP (100 μM, 150 μM, 200 μM) which were below its IC50 value (HN6: 224.6 μM, Cal27: 219.8 μM, SCC9: 210 μM) and DDP (2 μg/mL, 4 μg/mL, 6 μg/mL) for a total of 24 h treatment. As shown in Fig. 3 (a, c, e), CAP combined with DDP induced a significant inhibitory effect in the three TSCC cell lines compared to CAP or DDP alone. We used Chou-Talalay isobologram analysis (Calcusyn Graphing Software Version 2.11, Biosoft Inc., Ferguson, MO, USA)^31^ to analyze whether CAP combined with DDP has the potential to synergistically enhance cytotoxicity in TSCC cells. As previously demonstrated, synergy was indicated when the confidence interval (CI) is < 1. The results of the CI for different concentrations of CAP and DDP in the three TSCC cell lines are shown in Fig. 3 (b, d, f). The majority of combinations exhibited synergistic effects (CI < 1). These findings suggest that low-toxicity concentrations of CAP and DDP have synergistic inhibitory effects on TSCC cells in vitro. Consequently, we selected 150 μM CAP and 4 μg/mL DDP for subsequent experiments, as both displayed minimal toxicity when used individually and demonstrated remarkable synergistic outcomes when used together.

**Figure 4:**
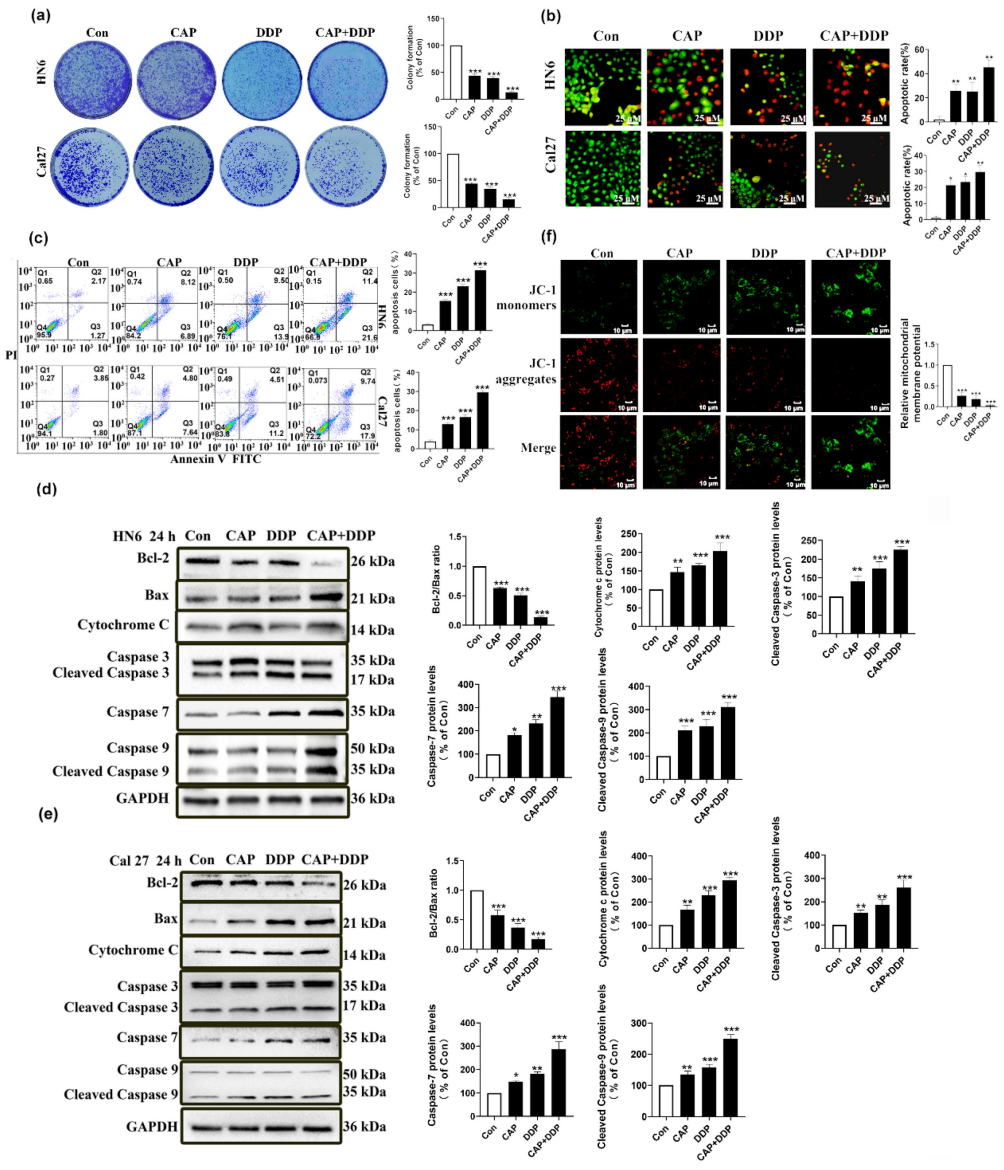
** Capsaicin (CAP) combined with Cisplatin (DDP) has synergistic anti-tumor effects in Tongue squamous cell carcinoma (TSCC) cells. (a)** Colony formation assay in HN6 and Cal27 cells. Data for the CAP (150 μM) and/or DDP (4 μg/mL) treatment groups were normalized and compared with the untreated control group. **(b, c)** The apoptotic effects of HN6 and Cal27 cells, after CAP (150 μM) and/or DDP (4 μg/mL) treatment, were detected by dual acridine orange (AO)/ethidium bromide (EB) fluorescent staining assay** (b),** and Annexin V-FITC/PI double staining **(c),** Bar = 25 μm. The percentage of apoptotic cells were detected by the number of Q2+Q3. **(d, e)** The expression of mitochondrial apoptosis pathway related protein, namely Bcl-2, Bax, Cytochrome c, and Caspase-3/-7/-9, were detected by Western blotting after CAP (150 μM) and/or DDP (4 μg/mL) treatment for 24 h in HN6 cells **(d)**, and Cal27 cells **(e)**. For normalization, Glyceraldehyde 3-phosphate dehydrogenase (GAPDH) was utilized. **(f)** The effect of CAP combined with DDP on changes in mitochondrial membrane potential (Δψm) of HN6 cells was determined by fluorescence microscopy using JC-1 staining. Bar = 10 μm. Data are presented as the Mean ± SD from three independent experiments. Statistical significance was indicated by *, *P* < 0.05; **, *P* < 0.01; and ***, *P* < 0.001.

In addition, colony formation experiments demonstrated that the combination of CAP and DDP resulted in a significant reduction in the number of cell clones in HN6 and Cal27 cells compared to the use of CAP or DDP alone (Fig. 4a). This suggests that CAP and DDP synergistically inhibited the proliferation of TSCC cells. Additionally, the AO/EB double fluorescence and Annexin V-FITC/PI double staining assays revealed that after 24 h of treatment, the combination of CAP and DDP led to a significant increase in the number of apoptotic HN6 and Cal27 cells compared to the use of CAP or DDP alone (Fig. 4b-c). These results indicate that CAP and DDP synergistically induce apoptosis in TSCC cells.

In summary, our experimental findings demonstrated that the combination of CAP and DDP exhibited a synergistic anti-tumor effect in TSCC.

### Capsaicin combined with cisplatin can induce apoptosis of TSCC cells through the mitochondrial pathway

To verify whether the apoptotic effect induced by CAP and\or DDP in TSCC cells was via the mitochondrial apoptosis pathway, tongue cancer cells (HN6, Cal27) were treated with CAP (150 µM) and/or DDP (4 μg/mL) for 24 h, and the pro-apoptotic effect was verified by the change in the level of mitochondrial apoptosis-related proteins (Fig. 4d-e), which indicated that CAP combined with DDP could enhance mitochondria-mediated apoptosis.

During apoptosis, the depolarization of mitochondrial transmembrane potential (Δψm) indicated the disruption of mitochondrial membranes, and mitochondrial depolarization is manifested by a reduction in the ratio of the red-green fluorescence intensity by the JC-1 assay 32. ​We previously demonstrated that CAP treatment can disrupt the functional morphology of mitochondria by altering their ultrastructure. Hence, we further aimed to use the fluorescent probe JC-1 to measure whether there were any changes of intracellular Δψm after CAP (150 µM) and/or DDP (4 μg/mL) treatment. Based on the data presented in Fig. 4f, it is evident that the extent of mitochondrial membrane potential impairment was significantly higher in the group treated with CAP and/or DDP, particularly in the combined group. In this group, JC-1 existed predominantly in its monomeric form, with almost negligible formation of the JC-1 complex, in contrast to the groups treated with either CAP or DDP alone.

In summary, these results show that CAP combined with DDP induces apoptosis in TSCC cells through the mitochondrial pathway.

### CAP synergizes with DDP enhances mitochondrial apoptosis of TSCC cells via the TRPV1 receptor and the Calpain pathway

**Figure 5:**
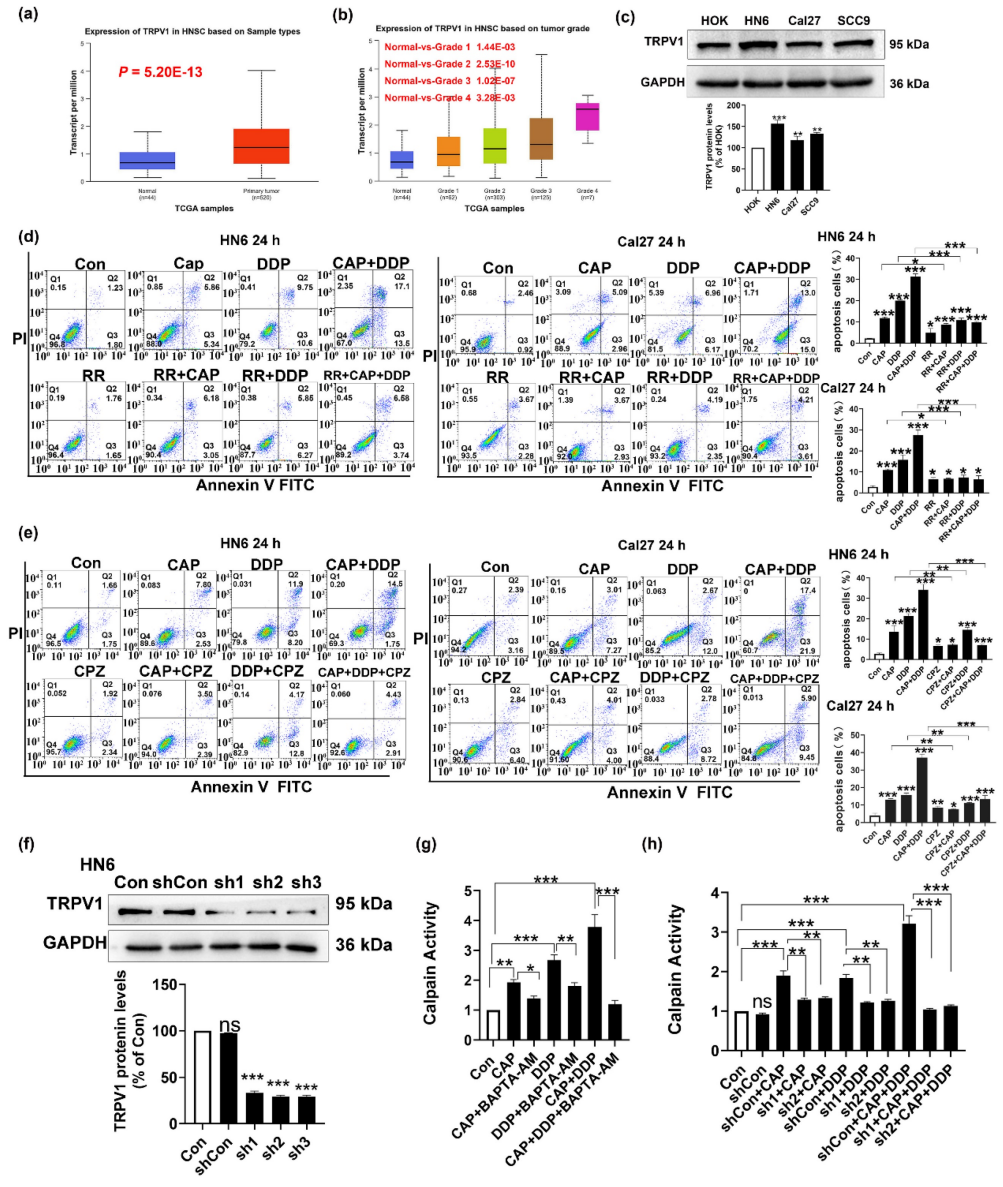
** Capsaicin (CAP) synergizes with Cisplatin (DDP) induced mitochondrial apoptosis in Tongue squamous cell carcinoma (TSCC) via transient receptor potential vanilloid 1 (TRPV1) receptor and the Calpain Pathway. (a, b)** TRPV1 messenger RNA expression in head and neck squamous cell carcinoma based on sample types **(a)**, tumor grade** (b)** in TCGA database. **(c)** Protein expression of TRPV1 in HOK, HN6, Cal27 and SCC9 cells. Glyceraldehyde 3-phosphate dehydrogenase (GAPDH) served as the normalized control. **(d)** Annexin V-FITC/PI double staining showed that Ruthenium red (RR), a TRPV generalized antagonist, inhibits apoptosis induced by CAP and/or DDP in HN6 and Cal27 cells. **(e)** Capsazepine (CPZ), an antagonist of TRPV1 receptor, inhibits the pro-apoptotic effect induced by CAP and/or DDP in HN6 and Cal27 cells. **(f)** The protein expression of TRPV1 in HN6 cells that were transfected with short hairpin RNAs (shRNAs, sh1, sh2, and sh3). Glyceraldehyde 3-phosphate dehydrogenase (GAPDH) served as the normalized control. **(g, h)** Human calpain enzyme-linked immunosorbent assay (ELISA) showed the calpain activity after pretreated with BAPTA-AM (10 μM), a well-known membrane permeable Ca^2+^ chelator **(g)**, and in TRPV1 stable knockdown cell lines (sh1 and sh2) after CAP and/or DDP treatment **(h)**. Data are presented as the Mean ± SD derived from biological triplicates. ns, no significance; *, *P* < 0.05; **, *P* < 0.01; ***, *P* < 0.001.

CAP is a well-known receptor agonist of TRPV1; when activated by CAP, TRPV1 can lead to an excessive influx of intracellular Na^+^ and Ca^2+^ resulting in cell death 18-20. To verify whether the apoptotic effect induced by CAP and/or DDP was mediated by the TRPV1 receptor, we first used TCGA database to detect the mRNA expression of TRPV1 in HNSC. As shown in Fig. 5a, TRPV1 was highly expressed in primary tumors compared to that in normal tissues (P = 5.20 E-13). Furthermore, there was a noticeable increase in the expression of TRPV1 across all HNSC tumor grades (Fig. 5b). Moreover, the expression of TRPV1 in the three tongue cancer cell lines (HN6, Cal27, and SCC9) was significantly higher than that in normal oral keratinocytes HOK, with HN6 cells showing the most pronounced difference (Fig. 5c). These results suggested that TRPV1 is overexpressed in TSCC, making cells with elevated TRPV1 levels more susceptible to intracellular calcium overload and subsequent cell death upon external stimulation and activation than cells with low or no TRPV1 expression.

Furthermore, to investigate whether apoptosis induced by CAP and/or DDP was associated with the TRPV1 receptor, we used the Annexin V-FITC/PI double staining assay to conduct a stepwise verification process. Initially, two tongue cancer cell lines (HN6 and Cal27) were pre-treated with ruthenium red (RR), a TRPV generalized antagonist, and then co-treated with CAP and/or DDP for 24 h. The number of apoptotic cells presented a significant reduced in both the single- and combined-drug groups after pretreatment with RR. ​Notably, the CAP + DDP group showed a more pronounced effect, suggesting that CAP and/or DDP induced apoptosis via the TRPV channels (Fig. 5d). Next, CPZ, an antagonist of TRPV1, was used. These results showed that CPZ pretreatment inhibited the pro-apoptotic effects of CAP and DDP in tongue cancer cells. Additionally, the CAP and DDP combination group displayed a more pronounced effect, suggesting that the pro-apoptotic effects of CAP and/or DDP were mediated via the TRPV1 receptor (Fig. 5e).

Moreover, we constructed a TRPV1 stable knockdown cell lines in HN6 cells through lentivirus and by using western blotting assay, we demonstrated that the three TRPV1 stable knockdown cell lines (sh1, sh2, sh3) exhibit significant knockdown effects compared to the vehicle group. The growth rates of sh1 and sh2 cells were faster than those of sh3 cells, and the knockdown effect was also evident. Therefore, sh1 and sh2 were selected for subsequent experiments (Fig. 5f).

Human calpains, Ca^2+^-activated cysteine proteases, are associated with tumor progression 33. In addition, calpain activation induced by Ca^2+^ influx promotes the apoptotic signaling cascade in tumor cells 34. Therefore, we used the Ca^2+^ chelator BAPTA-AM to pretreat HN6 cells. Next, the cells were co-treated with CAP and/or DDP for 24 h. Our findings revealed that CAP and DDP significantly activated calpain activity, with a more pronounced effect observed in the combination group. However, a marked reduction in calpain activity was observed after BAPTA-AM treatment. These results suggested that the combination of CAP and/or DDP could activate calpain activity through intracellular Ca^2+^ overload (Fig. 5g). Subsequently, to verify whether CAP combined with DDP caused intracellular Ca^2+^ overload through the TRPV1 receptor, and thereby activated calpain activity, we treated HN6 cells and shTRPV1 stable knockdown cell lines (sh1 and sh2) for 24 h with CAP and/or DDP. The results revealed that knockdown of TRPV1 resulted in a notable reduction in calpain activity after CAP and/or DDP treatment (Fig. 5h).

These results provide evidence that CAP synergizes with DDP, leading to intracellular Ca^2+^ overload via the TRPV1 receptor, subsequently activating calpain activity.

### CAP combined with DDP inhibits tumor growth in human TSCC xenograft model

**Figure 6:**
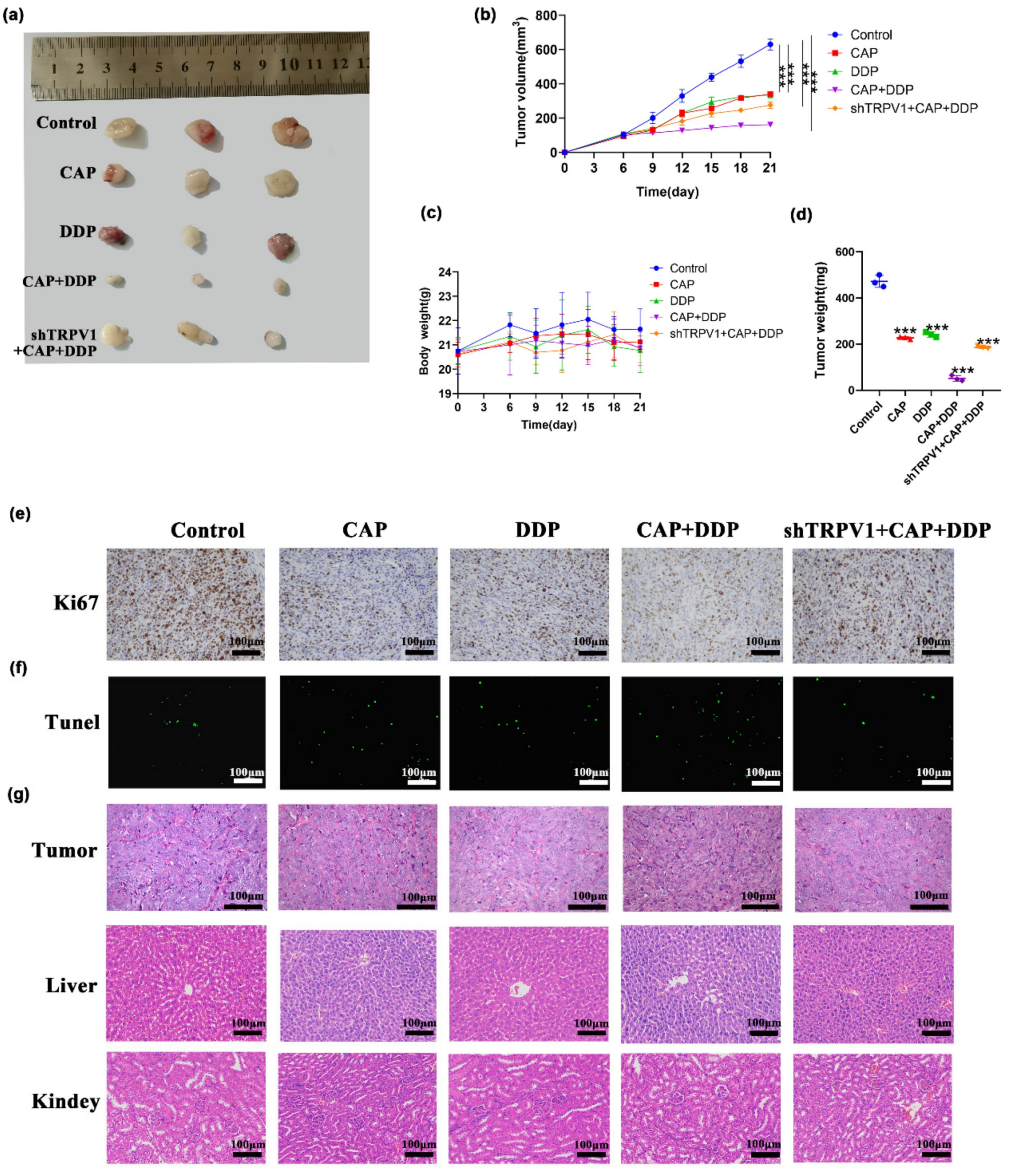
** Capsaicin (CAP) synergizes with Cisplatin (DDP) inhibited tumor growth in human Tongue squamous cell carcinoma (TSCC) xenograft model.** Effect of CAP combined with DDP on the growth of human TSCC xenograft tumors was studied. A total of twenty-five nude mice received CAP: 10 mg/kg and/or DDP: 4 mg/kg by gavage every three days. After the final treatment, the mice were sacrificed, and subcutaneous xenograft tumors were collected. **(a)** Images of the tumors were captured, **(b, c)** and xenograft tumor volumes as well as body weight of each nude mice were measured at designated time points. **(d)** Additionally, tumor weight was assessed after CAP and/or DDP treatment**. (e, f)** Sections of the xenograft tumors were prepared and subjected to immunohistochemical staining with Ki67** (e)** and TUNEL** (f)** to assess intratumor proliferation and apoptosis, respectively. **(g)** The histopathological changes in the tumor tissues, liver and kidney of nude mice by HE staining after CAP and/or DDP treatment. Quantitative data are shown as Mean ± SD of 5 independent experiments; *, *P* < 0.05; **, *P* < 0.01; ***, *P* < 0.001.

Based on our *in vitro* experiments showing that CAP and/or DDP exert anti-tumor effects via TRPV1 in TSCC cells, we further investigated whether they had the same inhibitory effect *in vivo* in a xenograft model. Nude mice were subcutaneously injected with HN6 and shTRPV1 stable knockdown cell lines in the right axilla. The mice were randomly divided into 5 groups: control group (200 μL PBS, gavage), CAP alone treatment group (10 mg/kg, gavage), DDP alone treatment group (4 mg/kg, gavage), CAP/DDP combination treatment group (CAP: 10 mg/kg + DDP: 4 mg/kg, gavage), and shTRPV1 stable knockdown cell lines + CAP / DDP combination treatment group (CAP: 10 mg/kg + DDP: 4 mg/kg, gavage). The treatments were administered once every 3 days, and the body weight and tumor volume of the mice were measured for 21 days. As shown in (Fig. 6a-d), compared with the control group, both drugs significantly reduced tumor volume and weight when used alone and in combination, with the combination treatment group showing the most pronounced inhibitory effect. In addition, the tumor volume and weight in the shTRPV1 + CAP + DDP combination treatment group were significantly larger than those in the non-knockdown combination group, indicating that CAP combined with DDP induced apoptosis through the TRPV1 receptor *in vivo*. There was no significant difference in the body weights of the mice between the control and treatment groups. Furthermore, the level of Ki67, a proliferation marker, was significantly reduced in the combination group. Conversely, in the group inoculated with shTRPV1 cells, the expression of Ki67 was significantly increased (Fig. 6e). Moreover, the TUNEL assay results confirmed the TRPV1-mediated inhibitory effect of CAP and DDP, which revealed a significant increase in the number of apoptotic cells in the drug-treated group, especially in the combination group. However, the number of apoptotic cells in nude mice inoculated with shTRPV1 cells showed a significant decrease (Fig. 6f). Finally, HE staining revealed no significant histopathological or cytological improvement in tumor tissues after CAP and/or DDP treatment. Nevertheless, it is noteworthy that there were no evident histopathological injuries in the liver and kidney tissues of the mice treated with CAP and/or DDP, indicating that the combination of CAP and DDP did not result in toxic side effects *in vivo* (Fig. 6g).

Overall, our findings indicate that the combined treatment with CAP and DDP induced notable apoptotic effects through TRPV1 receptors *in vivo* without any apparent toxic side effects.

## Discussion

DDP is commonly used as a primary chemotherapeutic drug for the treatment of TSCC 7. However, the efficacy of DDP is hindered by drug resistance and undesirable side effects. Therefore, it is crucial to identify potential drugs that exhibit low toxicity and enhance the sensitivity of DDP. Recently, several studies have highlighted the potential of natural dietary phytochemicals to inhibit HNSC, either alone or in combination with traditional chemotherapy drugs 35. For instance, biochanin A increases the expression of the death ligand FasL in FaDu cells in a dose-dependent manner, leading to apoptosis in HNSC 36. Similarly, 10 μM apigenin induces apoptosis by reducing the expression of Bcl-2 in SCC25 cells, activating caspase-3, and synergistically enhancing the sensitivity of 5-Fu and DDP in TSCC cells 37. Natural phytochemicals have been shown to ameliorate DDP-induced drug toxicity 38. CAP is the main pungent ingredient in chili peppers and has attracted wide attention in recent years owing to its notable anticancer effects 39. Our study revealed that CAP effectively inhibited TSCC cell proliferation in a dose- and time-dependent manner. CAP was found to alleviate DDP resistance in stomach cancer cells, indicating that it may be beneficial as an adjunct to DDP therapy 39, 40. Therefore, we investigated the effects of CAP and DDP on TSCC cells. Our results demonstrated that both CAP and DDP inhibited TSCC cell viability in a dose-dependent manner and that the combined use of subtoxic concentrations of CAP and DDP exerted significant synergistic anti-tumor effects (CI < 1) in TSCC cells. These results indicated that CAP enhanced the sensitivity of TSCC cells to DDP.

Apoptosis is an important barrier preventing cancer development. The mitochondrial pathway is the major focus of apoptosis research. It primarily involves altering the permeability of the mitochondrial membrane by increasing the intracellular Ca^2+^ concentration, releasing Cyt c, and activating caspase, a cysteine aspartic acid-specific protease, to induce apoptosis 41-43. CAP has been confirmed to induce apoptosis in non-small cell lung cancer 11, gastric cancer 15 and breast cancer 44. In this study, we explored the apoptotic effect of CAP on TSCC cells and demonstrated that CAP induced apoptosis in TSCC cells in a dose-dependent manner. Moreover, CAP treatment disrupted the functional structure of mitochondria in TSCC cells, leading to the disappearance of ruptured spines in the mitochondrial membrane. Furthermore, this disruption triggers the activation of proteins associated with the mitochondrial apoptosis pathway, downregulating the expression of the anti-apoptotic protein Bcl-2, upregulating the expression of the pro-apoptotic protein Bax, releasing Cyt c, and activating caspase-3,-7, and -9. CAP enhances DDP-induced apoptosis in human gastric cancer cells 14. In the present study, we also confirmed the combination of low toxic concentrations of CAP (150 μM) and DDP (4 μg/mL) significantly induced the apoptosis of TSCC cells, comparing with the effects of each drug alone. Previous studies have found that CAP treatment can induce mitochondrial pathway apoptosis in colon cancer cells by producing reactive oxygen species and destroying Δψm 22. DDP combined with α-Hederin induced the accumulation of ROS, reduced Δψm, and induced gastric cancer cells apoptosis in the mitochondrial pathway 45. Our results are consistent with those of previous studies and further show that the combination of CAP and DDP induces apoptosis via the mitochondrial pathway. In comparison to the control group, the CAP/DDP group showed a significant decrease in Δψm, accompanied by a reduction in Bcl-2, an increase in Bax, the release of Cyt c and the activation of capsase-3/7/9. In summary, these results indicate that combined treatment with low concentrations of CAP and DDP can effectively induce mitochondrial apoptosis in TSCC cells.

Ca^2+^ functions as a pivotal second messenger in cellular signaling, and changes in its intracellular concentration play a decisive role in determining cell fate and mediating the balance between cell apoptosis and proliferation 46. As a high-affinity receptor for CAP, TRPV1 has very high permeability to Ca^2+^. TRPV1-mediated cell death also appears to be related to mitochondrial dysfunction following Ca^2+^ influx 47. For instance, CAP induces apoptosis caused to mitochondrial calcium overload in anaplastic thyroid cancer cells through TRPV1 receptors 48. The combined use of DDP and selenium can induce oxidative stress and increase Ca^2+^ concentration in breast cancer cells MCF-7 by regulating TRPV1 channels, thereby exerting anticancer effects 49. In our study, we revealed that the expression of TRPV1 in normal oral epithelial cells was considerably lower than in the three TSCC cell lines. Additionally, our findings indicate that the use of the TRPV broad-spectrum inhibitor RR and the TRPV1 inhibitor CPZ significantly reduced the number of apoptotic cells. These results suggest that apoptosis induced by the combination of CAP and DDP in TSCC is mediated through the TRPV1 receptor, and that the discrepancy in expression levels could potentially serve as a novel starting point for developing TRPV1-based therapeutic interventions for TSCC.

Calpains are a family of Ca^2+^-dependent intracellular cysteine ​​proteases that regulate diverse cellular processes, including cell division, differentiation, migration, and apoptosis 50. Calpain is an important regulator of CAP-induced apoptosis 11. A previous study revealed that the combined apoptotic effect of CAP and camptothecin in human small-cell lung cancer was facilitated by the elevation of intracellular Ca^2+^ levels and activation of the calpain pathway 51. In this study, we discovered that the combination of CAP and DDP effectively triggered calpain expression in TSCC cells. Notably, this effect was more pronounced when both drugs were used together. However, we observed a significant inhibition of calpain activity when TSCC cells were pre-treated with the Ca^2+^ chelator BAPTA-AM, indicating that the combined use of CAP and DDP activates calpain activity in TSCC cells by elevating intracellular Ca^2+^ levels. These results are consistent with those of previous studies showing that CAP and DDP can increase intracellular Ca^2+^ concentration, and that activation of calpain activity can be mediated by Ca^2+^ overload 50, 51. Furthermore, TRPV1 knockdown led to a significant attenuation of calpain activity induced by CAP and/or DDP, indicating that the activation of calpain activity induced by the combination of CAP and DDP was mediated by TRPV1. In addition, we demonstrated that the anti-tumor effect of CAP combined with DDP *in vivo* was mediated through the TRPV1 receptor, which is consistent with the results of *in vitro*. Furthermore, the combined treatment with the two drugs had no significant effect on mouse body weight and no significant histopathological changes in the liver and kidney. These results suggest that CAP can be safely and effectively used to enhance the anti-tumor effects of DDP in TSCC.

However, this study has some limitations. First, the current studies have only examined the impact of TRPV1 knockdown and inhibiting Ca^2+^ on the induction of apoptosis by CAP and/or DDP in TSCC, neglecting the binding effects between CAP, DDP, and TRPV1 in TSCC. Second, identifying DDP-resistant targets, CAP-DDP-binding targets, and relevant intracellular signaling pathways through transcriptome sequencing or single-cell sequencing could be a promising avenue for future studies on TSCC treatment.

**Figure 7:**
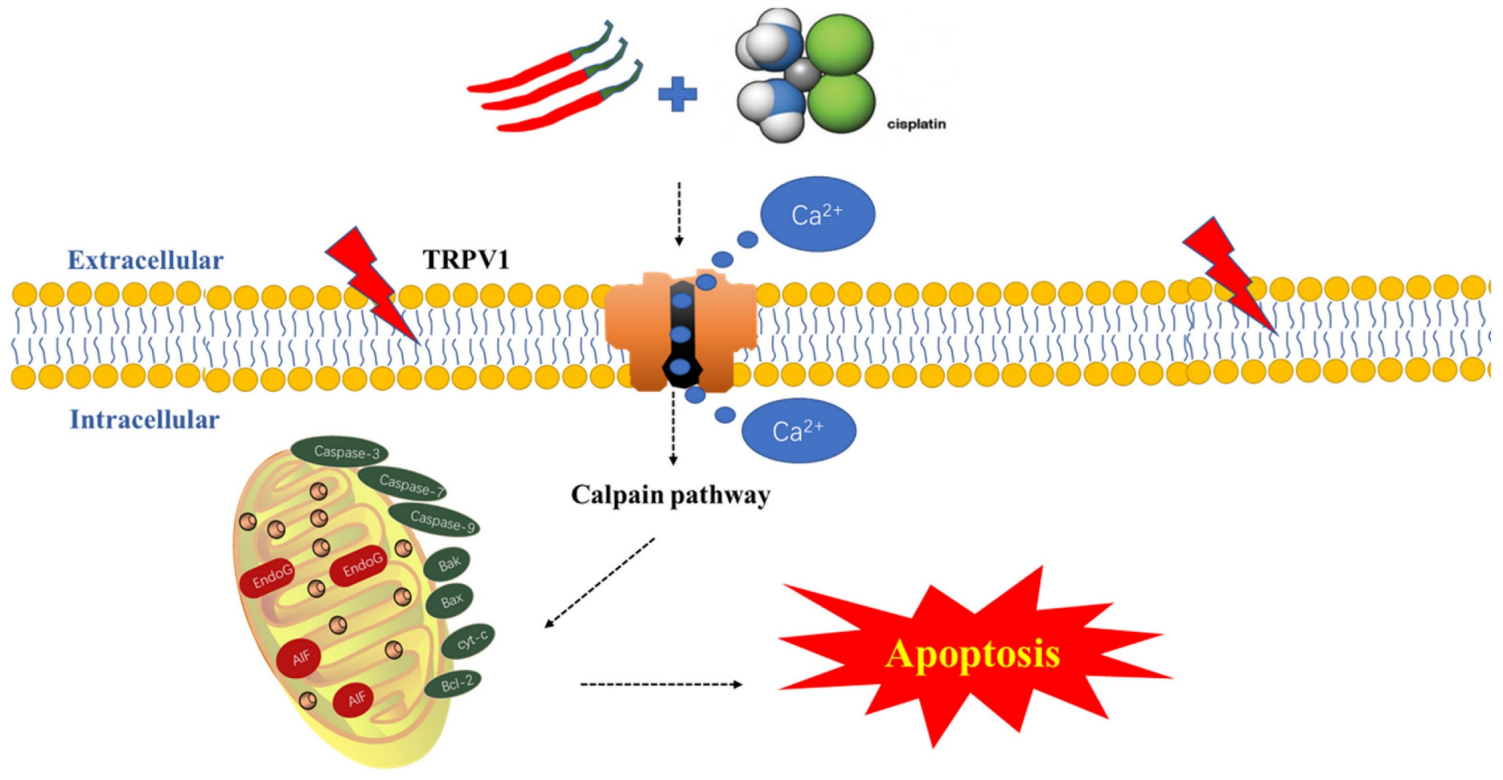
** Schematic diagram of the potential antitumor mechanism of Capsaicin (CAP) synergizes with Cisplatin (DDP) in tongue squamous cell carcinoma (TSCC) cells.** The combination of CAP and DDP activates the TRPV1 receptor, leading to an increase in intracellular Ca^2+^ concentration and activation of calpain activity. This, in turn, destroying the functional structure of mitochondria, reducing the mitochondrial membrane potential, releasing Cytochrome c, activating caspase 3/7/9, and downregulating anti-apoptosis protein Bcl-2, upregulating the pro-apoptotic protein Bax. These events ultimately induce mitochondrial pathway apoptosis. This cascade reaction reveals the potential mechanism by which CAP synergizes with DDP to induce apoptosis in TSCC via the TRPV1 receptor and calpain pathway.

In conclusion, for the first time, we revealed that CAP selectively inhibits TSCC cells, while exhibiting no apparent drug toxicity in normal oral cells. Additionally, CAP enhanced the sensitivity of TSCC cells to DDP, resulting in synergistic anti-tumor effects both *in vivo* and *in vitro*. Furthermore, we found that this mechanism is mediated by the calpain pathway, which is activated by TRPV1 receptor-mediated Ca^2+^ overload and calpain pathways (Fig. 7). Consequently, our study offers new potential adjunctive therapies for TSCC treatment, with the benefit of reducing the toxic side effects associated with traditional chemotherapy.

## Abbreviations

CAP: Capsaicin (trans-8-methyl-N-vanillyl-6-nonenamide)
DDP: Cisplatin (cis-diamminedichloroplatinum II)
TSCC: Tongue squamous cell carcinoma
OSCC: Oral squamous cell carcinoma
TRPV1: transient receptor potential vanilloid 1
RR: Ruthenium red
CPZ: Capsazepine
HOK: normal oral cells
